# Does Allostatic Load in 50–89‐Year‐Olds Predict the Development of Frailty? Evidence From a National Longitudinal Study Over 12 Years

**DOI:** 10.1002/smi.70219

**Published:** 2026-09-08

**Authors:** Marco Arkesteijn, Rachel Bennett, Jennifer L. Davies, Rachel C. Sumner

**Affiliations:** ^1^ Department of Life Sciences Aberystwyth University Aberystwyth UK; ^2^ Education & Applied Sciences University of Gloucestershire Cheltenham UK; ^3^ School of Healthcare Sciences Cardiff University Cardiff UK; ^4^ Translational Health Sciences Bristol Medical School University of Bristol Bristol UK

**Keywords:** ageing populations, allostatic overload, healthy ageing, registered report

## Abstract

Frailty is characterised by a loss of function across several domains but is not an inevitable aspect of ageing and can be reversed with intervention. Determining those who are more likely to become frail before physical deficits become manifest will allow earlier intervention. One promising indicator of the potential for frailty is allostatic load, a physiological status associated with prolonged stress that is characterised by multisystem dysfunction. Previous research has sought to understand the links between allostatic load and frailty, but has not yet explored whether allostatic load may be a predictive factor at younger ages. The present study examined whether allostatic load can be used as a predictive indicator of frailty. Using data from four waves of the English Longitudinal Survey on Ageing (ELSA) collected over 12 years with a sample of 3248 people between 50 and 89 years old at baseline, discrete time event history analysis was used to determine if allostatic load is associated with the onset of pre‐frailty and frailty. The findings indicate that allostatic load was a significant contributor to the occurrence of (pre‐)frailty. Age and sex did not interact with allostatic load and frailty predictions, whilst in particular social support was identified as a possible modifiable factor. Allostatic load determination should be considered as a possible screening tool for future frailty risk in currently non‐frail individuals.

## 1 Introduction

Ageing is associated with a progressive decline in physiological functions and metabolic processes (López‐Otín et al. 2023). This can result in frailty, an age‐related condition that is characterised by a decline in functioning across multiple physiological systems. Frailty is associated with higher health care costs, increased dependence, and lower quality of life (Kojima et al. 2016). Frailty can be prevented and, once present, reversed (Dent, Morley, et al. 2019; Negm et al. 2019; Travers et al. 2019), but prevention appears to be more (cost) effective (Apóstolo et al. 2018; Dent, Martin, et al. 2019; Looman et al. 2019). Early identification of those on the trajectory towards frailty would therefore be highly valuable (Walsh et al. 2023).

Allostatic load is characterised by multisystem dysfunction resulting from the physiological wear and tear incurred from chronic stress. Although both frailty and allostatic load reflect dysfunction in physiological systems, allostatic load is resultant from prolonged stress and is therefore identifiable at any point during the life course (Crimmins et al. 2003), whereas frailty is generally identifiable in later life only. Prolonged allostatic load has been associated with declines in cognitive and physical functioning over time (Seeman et al. 2001), as well as age‐related degenerative conditions such as dementia (Twait et al. 2023) and Alzheimer's disease (Matos and De Souza‐Talarico 2019), providing a link between allostatic load and adverse outcomes in ageing. Several shared pathophysiological processes between allostatic load and frailty exist, including dysregulation to endocrine and immune systems, along with impaired or dysfunctional signalling in brain areas that also process psychological stress and initiate the physiological stress response (Clegg et al. 2013), suggesting a potential link between these two phenomena. As allostatic load can be present at any point during the life span, and is associated with dysregulations and dysfunctions of several systems also implicated in frailty, this might suggest that allostatic load could contribute to the development frailty, and therefore be useful as a prognostic indicator (Kuchel 2009). Moreover, as allostatic load is reversible through a variety of interventions aimed at reducing overall psychological and physiological stress (Guidi et al. 2020), it is ameliorable and therefore may also be an intervention point to delay onset of frailty.

Allostatic load reflects the accumulation of stressors across the life course (Crimmins et al. 2003) and frailty reflects the resilience to stressors and its impact on functioning in older age (Dent, Morley, et al. 2019). Despite the evidence for the multi‐system impact of allostatic load, and some of its relations with later age outcomes, remarkably little research has been conducted to understand if, how, and when allostatic load may predict the onset of frailty. There is evidence from cross‐sectional studies that allostatic load may be associated with earlier development of frailty (Fried et al. 2009; Guidi et al. 2020; Szanton et al. 2009). A similar relation has been suggested based on longitudinal research (Ding et al. 2017; Karlamangla et al. 2002), however, the design of these studies limits the conclusions that can be made. Ding et al. did not exclude participants with frailty at baseline, and only included participants over the age of 65 years, whilst Karlamangla et al. (2002) only included participants aged between 70 and 79 years at baseline. Co‐existence of frailty and allostatic load is likely, particularly in older age categories, and it cannot be determined if allostatic load preceded frailty. To establish whether allostatic load precedes the development of frailty in later life, a baseline with participants without frailty is warranted. Previous research (Shi et al. 2023) has also used overlapping concepts of allostatic load and frailty, by employing the frailty index, rather than frailty phenotype. Use of the frailty index means that similar, or even the same, variables will be used to quantify frailty and allostatic load as both rely on establishing the ratio of presence or absence of a (medically diagnosed) self‐selected range of conditions (Cesari et al. 2014). In contrast, the frailty phenotype emphasises five domains of physical functioning (weight loss, weakness, exhaustion, slowness and low physical activity) specifically and exclusively (Cesari et al. 2014; Fried et al. 2001). Combined, this indicates it is currently unknown whether allostatic load is relevant as a prognostic indicator of frailty. Establishing such a relation may facilitate early identification of individuals who would benefit the most from interventions to prevent frailty, rather than these individuals only being identified when the decline in functioning becomes noticeable.

In summary, an association has been suggested between allostatic load and frailty both cross‐sectionally (Fried et al. 2009; Guidi et al. 2020; Szanton et al. 2009) and longitudinally (Ding et al. 2017; Karlamangla et al. 2002), however it is still not known whether allostatic load precedes the onset of frailty, and if it can predict frailty onset. Therefore, the aim of this research was to determine, among non‐frail individuals, if allostatic load is a predictor of future frailty. Using the existing dataset of the English Longitudinal Study of Ageing (ELSA), allostatic load was determined at baseline and frailty was evaluated every 4 years thereafter for 12 years. It was hypothesised that allostatic load at baseline would be associated with a greater risk of developing frailty, and this relation would be present in adults who are young‐old (50–64 years) at baseline as well as those who are older (65–89 years) at baseline.

## 2 Methodology

This study was conducted as a Registered Report, with methods and analysis defined and peer reviewed prior to the commencement of the study. The timeline included submission of the Stage 1 Registered Report in November 2023, approval in November 2024 and completion of project in December 2025. The approved protocol was registered on the Open Science Framework repository in December 2024 (https://osf.io/5chvn).

### 2.1 Database Selection

Candidate longitudinal population survey datasets were identified using the Gateway to Global Ageing Data (Gateway to Global Ageing Data 2023), Google Scholar, and an initial scoping search of published literature on allostatic load or frailty. For consideration for inclusion in this study, a dataset had to meet the following criteria: At least 10 years of follow‐up; contain biomarker data; contain data sufficient to establish (pre)frailty; include participants aged 50–100 years; data stored in English. Thirty‐four datasets were identified and scrutinised to determine whether they incorporated variables that would enable frailty and allostatic load to be calculated, with the former preferentially based on functioning and the latter based on physiological biomarkers. Three datasets incorporated data that could be used to measure both the frailty phenotype model and allostatic load based on physiological biomarkers: ELSA, The Irish Longitudinal Study on Ageing (TILDA) and the Health and Retirement Study (HRS). At the time of writing the Stage 1 Registered Report for this study (November 2023) access to the TILDA dataset was suspended, with no projected date of availability, so this database was excluded from consideration. HRS and ELSA were both deemed appropriate to address the research question, containing data from United States of America and England, respectively. ELSA was selected for the study, as the authors are based in the United Kingdom and have better understanding of socio‐demographic impacts and relevant co‐variates in the UK context than in the USA context.

### 2.2 Overview of Study

This longitudinal study used secondary survey data from ELSA collected over 12 years (2004–2016). Allostatic load was determined for a population of non‐frail individuals using physiological biomarkers obtained in 2004/2005. Frailty status (non‐frail: 0 out of 5 indicators, pre‐frail: 1 or 2 indicators, frail: 3 or more indicators) was determined using data from every available timepoint thereafter. The progression from non‐frail to pre‐frail and from non‐frail to frail was evaluated using discrete time event history analysis models.

### 2.3 Study Population

Participants in ELSA comprise a representative sample of people living in England aged 50–90+ years (Banks et al. 2021). Data have been collected in 10 waves spanning from 2002 to 2022. The earliest time point at which biomarkers were collected was Wave 2 (2004/2005). This formed the baseline timepoint of the current analysis. Variables required to evaluate frailty were also collected in Wave 2 and then subsequently in Wave 4 (2008/2009), Wave 6 (2012/2013), Wave 8 (2016/2017), whilst Wave 10 did not include grip strength (2020/21). In these Waves, participants were followed up by computer‐assisted personal interviews and self‐completed questionnaires as well as a health examination nurse visit for collecting medical information. Individuals were excluded from the analysis for this study if they were aged 90 years and older at Wave 2 because their age is top coded as ‘90’, if they had any frailty phenotype indicator at Wave 2, or if there were missing data for any of the variables required to determine allostatic load at Wave 2. All respondents gave informed consent as part of ELSA and consented to their data being used for secondary analyses.

### 2.4 Sample Size Estimation

It was not possible to know the available sample size until the data were accessed, therefore the sample size could only be estimated, rather than calculated precisely, at the time of preparing the Stage 1 Registered Report. The anticipated sample size available at baseline was larger than 2541 non‐frail participants, as this is the number of participants included in an existing analysis of wave 4, and it is likely that some participants were lost to follow up between wave 2 (the baseline for this study) and wave 4 (Leme and de Oliveira 2023). Attrition rate was unknown over this period of time, although Leme and de Oliveira (2023) indicated it was 39% between Waves 4 and 6.

The primary statistical analyses is a discrete time event history analysis model (also often called discrete time survival model). The study was guided by the common rule of thumb that there should be 10 events for every independent variable included in the model (Peduzzi et al. 1996). In this analysis there are 12 independent variables (allostatic load plus 11 covariates explained in a subsequent section). This requires at least 120 [12 independent variables multiplied by 10 (Peduzzi et al. 1996)] participants at baseline to go onto experience the outcome event.

### 2.5 Frailty Definition

The two main approaches used to identify frailty comprise the relative presence of ageing‐related health conditions accumulated deficits, or the frailty phenotype (Cesari et al. 2014). The frailty phenotype model reflects the robustness and resilience of an individual's functioning and was selected for this study. The frailty phenotype reflects five indicators of daily functioning, comprising low gait speed, weak grip strength, low physical activity level, exhaustion, and unintended weight loss (Fried et al. 2001). A person exhibiting three or more indicators is considered to have frailty, whereas one or two indicators is classified as pre‐frailty. Compared with the original phenotype model (Fried et al. 2001), four variables in ELSA are identical (low gait speed, weak grip strength, exhaustion, and unintended weight loss) and one had to be adapted (low physical activity level).

Exhaustion was derived from responses to two individual questions within the Centre for Epidemiologic Studies Depression Scale (CES‐D: Radloff 1977). The CES‐D is a 20‐item self‐report measure where respondents rate how often over the past week they experienced each listed symptom on a Likert scale ranging from zero (rarely or none of the time) to three (most or almost all the time). Exhaustion is considered present if a positive answer (‘Yes’) was provided to either of these two questions: ‘Felt that everything I did was an effort in the last week’ or ‘Could not get going in the last week’.

Gait speed was assessed only in participants aged 60 years and over by measuring the time taken to walk a distance of eight feet at usual pace (Veronese et al. 2017). Low gait speed is defined using the mean gait speed of two walks at normal pace with the sex and height cutoffs suggested by Fried et al. (2001), even though the recommended walk distance is 15 feet (and not eight feet). Any participant who could not perform the gait test owing to medical reasons was categorised as having low gait speed. Participants under the age of 60 years did not have their gait assessed and were assumed to not have a low gait speed. Thus, if this age group did not exhibit any of the other frailty phenotype criteria they were included.

Grip strength was measured using a handheld dynamometer. Participants were instructed to squeeze the dynamometer as hard as they could. Maximum grip strength was determined as the highest value recorded after three attempts with each hand. Weakness was defined using the sex and body mass index (BMI) cutoffs suggested by Fried et al. (2001), with actual cut off values determined based on the ELSA sample. Those not able to do the handgrip strength for medical reasons were considered as having weakness.

Weight change was defined as the difference in body mass (kg) between the current body weight relative to the body weight 4 years earlier (previous wave). Either the loss of ≥ 10% of body weight since previous time point or current BMI < 18.5 kg/m^2^ was defined as the cut off criteria (Gale et al. 2014).

Physical activity level was derived from a physical activity interview. Participants were asked about the frequency with which they did moderate or vigorous exercise (e.g., gardening, cleaning the car, walking at a moderate pace, dancing, floor or stretching exercise). These questions had four response options (more than once a week, once a week, one to three times a month, and hardly ever or never). Low physical activity was defined as a response of ‘hardly ever or never’ to both the moderate and vigorous exercise questions. This item was different than that proposed by Fried et al. (2001) because this set of criteria considers a different scale for defining low physical activity.

### 2.6 Allostatic Load Definition

A measure for allostatic load was created using biomarker data from multiple domains, using the quantification methods outlined by Seeman et al. (1997) and using the system approach outlined by Richards et al. (2023) to account for the likely impact of age‐related chronic illness on the various markers being assessed, and the inequity of number of markers across each system that they represent. The domains and markers that were used from the ELSA dataset are: immune system (C‐reactive protein [CRP], fibrinogen); metabolic system (total‐to‐HDL cholesterol ratio, triglycerides); cardiovascular system (systolic blood pressure [SBP] and diastolic blood pressure [DBP]), and body fat deposition (BMI). Glycated haemoglobin (Hba1c) was not used in these analyses due to a high degree of missingness within the dataset identified after the Registered Report. These seven markers were dichotomised into risk level (high or low) according to established criteria quartiles associated with overall risk generally, or with relevance to sex‐specific risk (Sanders et al. 2017). The allostatic load index was then created by summing all of the risk levels per marker, where ‘high’ was attributed a value of 1, and ‘low’ a value of 0, with higher total index scores indicating higher allostatic load. Each system was assigned an allostatic load risk score, where each ‘high’ or ‘low’ attribution was given equal weight, correcting for physical health conditions and medications that impact each system. This resulted in a scale of 0–4, where 0 indicates no presence of allostatic load risk, up to 4, indicating allostatic load risk present for each system.

### 2.7 Covariates

#### 2.7.1 Demographic

To account for differences associated with both physical longevity and differentials between allostatic load and frailty, participant sex and age were included. As allostatic load is understood to stabilise for a time around retirement (Juster et al. 2010), and age of retirement has also been found to be associated with frailty (Van der Elst et al. 2021), retirement was included as a covariate for the analyses.

#### 2.7.2 Socio‐Economic Status

Various aspects associated with socio‐economic status are known to contribute to overall stress across the life course. As allostatic load is an index of accumulated physiological dysregulation due to chronic stress, level of education and wealth were included as covariates associated with social status. Level of education was split into a binary category of either having some qualifications or no qualifications at all. Wealth was assessed by creating a binary categorisation of the non‐pension wealth deciles available within the dataset, with the bottom two deciles being grouped into a ‘low’ category and the other eight forming the ‘medium to high’ category.

#### 2.7.3 Health

Smoking behaviour, BMI, and consumption of alcohol were included as covariates due to their capacity to impact each of the systems being assessed within the allostatic load index. Consumption of alcohol was defined as the respondent reporting having an alcoholic drink less than three times a week in the last 12 months versus at least three times a week in the last 12 months. As participant height was not collected in Waves 8 and 10, BMI was calculated using Wave 2 height data in each BMI calculation. Cognitive function was also incorporated into the analysis as a covariate because its association with frailty and that cognition could be included within the frailty phenotype. Cognitive function was measured using a cognitive function index based on a combined memory and executive function test performance, with a score between 0 and 49 and where a higher score indicates better cognitive performance.

#### 2.7.4 Social Context

To account for the contribution of the participants' social context into their overall health, a variety of covariates capturing elements of individual's social proximity and interaction were used. Social integration was included as a means to assess levels of potential loneliness, a factor known to be strongly associated with both allostatic load and frailty (Juster et al. 2010; Kojima et al. 2016). Social integration was calculated using a combined score of five items: partnership status (living with spouse/partner or not); three measures of frequency of contact (with children, with family, with friends) by either physically meeting, speaking with on the telephone, or writing emails or letters; and whether or not they are members of a social club, or other similar type of organisation or society. Participants with no children, relatives, or friends were given the maximum score for poor social integration and social support (i.e., the same score as those who see their loved ones one or twice a year or less). Social support was determined similarly according to Ding et al.’s (2017) conceptualisation, combining both a lack of positive support from friends and family and the presence of negative support also. Lack of positive support was measured using any responses that indicate disagreement to questions in the self‐complete questionnaire module about partnerships and friendships, including ‘How much do they really understand the way you feel about things?’, ‘How much can you rely on them if you have a serious problem?’, and ‘How much can you open up to them if you need to talk about your worries?’. The presence of positive support was measured using responses that indicate agreement or high frequency to statements such as ‘How much do they let you down when you are counting on them?’ and ‘How much do they criticise you?’.

### 2.8 Statistical Analysis

Analyses were run separately to evaluate each of the two outcome variables: frailty and pre‐frailty. The sample for the analysis based on the pre‐frail outcome variable excluded frail individuals. Based on life table estimates, the survivor functions (often referred to as survivor curves) for each outcome by age group, sex and allostatic load were plotted as a function of time since baseline. This gives an overview of the proportion of the sample at baseline who remain non‐frail/non‐prefrail over time and differences in these proportions by age group, sex and allostatic load at baseline.

Discrete‐time event history models were then used to model the odds of experiencing frailty/pre‐frailty, whilst accounting for time since baseline. A discrete‐time approach was used because frailty/pre‐frailty was only measured once per survey wave, so the exact timing of becoming frail/pre‐frail is not known. Binary logistic regression modelling was performed on the data in person‐period format, where each observation represents a participant's data for each wave ‘at risk’ of experiencing the outcome.

In the first models, allostatic load and time were included as covariates to examine whether there is significant association between allostatic load at baseline and propensity to experience frailty/pre‐frailty after accounting for time only. In the subsequent models, demographic characteristics, socio‐economic characteristics, health characteristics and social support characteristics were added in turn alongside time to examine whether controlling for these factors changed the association between allostatic load and propensity to experience frailty/pre‐frailty. In the final models, time, allostatic load, demographic characteristics, social characteristics, health characteristics and social support characteristics were all included alongside time. Statistical significance was determined using an alpha level of 0.05.

This modelling approach was applied to the whole sample to explore the association between allostatic load and propensity to experience frailty/pre‐frailty amongst all 50–89‐year‐olds. In the Stage 1 Registered Report, we specified that the sample would then be stratified by age group and the modelling repeated for each age group separately (with the same covariates)‐ that is, firstly 50–59 year olds only, secondly 60–69 year olds only, thirdly 70–79 year olds only and lastly 80–89 year olds only. This was designed to reveal whether the predictive power of allostatic load for future frailty/pre‐frailty varies by age. As a robustness check, we also proposed to repeat the modelling for males and females separately (with the same covariates) to examine whether the predictive power of allostatic load for future frailty/pre‐frailty varied by sex. However, after viewing the data, this age and sex stratified analysis was deemed not feasible due to low number of events.

#### 2.8.1 Missing Data

To account for missing data in the covariates, cases where less than 5% of respondents had missing data were excluded, or if more than 5% had missing data, a ‘missing data’ category was created. An analysis of the characteristics of participants with and without missing data was conducted to describe any impacts of removing these cases on the representativeness of the sample. The data was then reshaped into person‐period form where each row represents an observation succeeding baseline and before the individual leaves the risk set—either by experiencing the outcome variable being study (frailty or pre‐frailty) or because they were lost to follow up.

## 3 Results

### 3.1 ELSA Sample Characteristics

A total of 3248 participants met the inclusion criteria at baseline (ELSA Wave 2): non‐frail and with complete data for all components of the quantification of allostatic load. Missing categories were included for social integration, social support and allostatic load as more than 5% of cases had missing data. Allostatic load data showed a high level of missingness, with 27% (*N* = 879) respondents having missing data. An analysis of the characteristics of dropped cases compared to non‐dropped cases revealed no significant associations with age, sex, education, wealth, smoker status, alcohol consumption, BMI or social support. However, there were significant associations with social integration and allostatic load: individuals with missing data for social integration and for allostatic load were also more likely to have missing data across other variables and thus dropped from the analysis.

As can be seen from Table 1, the mean age of the sample at baseline was under 70 years, with a slight majority female (*N* = 1720, 53%), not retired (*N* = 1800, 55%), a light consumer of alcohol (less than three times a week *N* = 1921, 59%), a strong majority educated (*N* = 2423, 75%), in medium‐to‐high income brackets (*N* = 2985, 92%), and not currently smoking (*N* = 2869, 88%). The participant pool provided a relatively even distribution of social connection, with a small majority of participants showing higher social integration (*N* = 1131, 35%) and social support (*N* = 1074, 33%).

**TABLE 1:** Sample characteristics at baseline.

	*N*	%	Mean	Standard deviation
Sex				
Male	1528	47		
Female	1720	53		
Age (years)			63	7.6
Retirement status				
Not retired	1800	55		
Retired	1448	45		
Education				
No qualifications	825	25		
Some qualifications	2423	75		
Wealth				
Low	263	8		
Medium‐high	2985	92		
Smoker				
Not a current smoker	2869	88		
Current smoker	379	12		
BMI			28	4.5
Alcohol consumption				
Less than 3 times a week	1921	59		
At least 3 times a week	1327	41		
Cognitive function			31	5.5
Social integration				
High	1131	35		
Medium	969	30		
Low	793	24		
Missing	355	11		
Social support				
High	1074	33		
Medium	1047	32		
Low	802	25		
Missing	325	10		
Allostatic Load				
0	458	14		
1	672	21		
2	699	22		
3	381	12		
4	159	5		
Missing	879	27		
Total	3248	100		

By the end of the 12‐year observation period (ELSA Wave 10), 186 incident cases (6%) had developed frailty (Table 2) and 1215 incident cases (37%) had developed pre‐frailty (Table 3). Of the 186 individuals who developed frailty, 91 (3% of the total sample) developed frailty without previously being classified as pre‐frail (Table 3). For the frailty analysis, cases lost to follow up or that did not complete all questions for frailty measures in follow‐up waves (ELSA Waves 4, 6, 8 and 10) were significantly older, more likely to have no qualifications, low wealth, be a smoker, have missing data on social support and have missing data or a score of 0 for allostatic load at baseline than cases with complete data for all frailty measures. There was no significant association with sex, BMI, alcohol consumption or social integration. For the prefrailty analysis, cases lost to follow up or that did not complete all questions for frailty measures in follow‐up waves were significantly younger, more likely to be male, have no qualifications, be a smoker and have missing data on social support at baseline. There was no significant association with wealth, BMI, alcohol consumption, social integration or allostatic load.

**TABLE 2:** Summary of outcomes by the end of the observation period for the frailty analysis.

	*N*	%
Not frail by the end of the observation period	1119	34
Frail before the end of the observation period	186	6
Lost to follow up or did not complete all questions for frailty measure	1943	60
Total	3248	100

**TABLE 3:** Summary of outcomes by the end of the observation period for the prefrailty analysis.

	*N*	%
Not prefrail by the end of the observation period	456	14
Prefrail before the end of the observation period	1215	37
Frail before the end of the observation period (with no measurement as prefrail)	91	3
Lost to follow up or did not complete all questions for frailty measure	1486	46
Total	3248	100

### 3.2 Survival Curves

**FIGURE 1:**
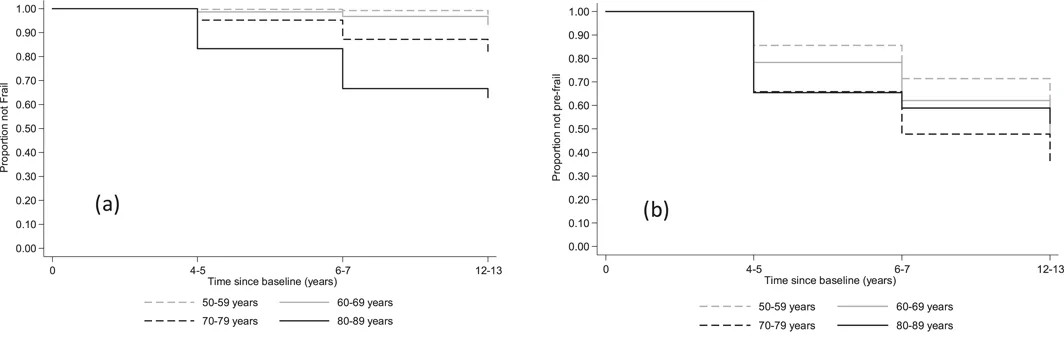
Frailty (1a) and pre‐frailty (1b) survival curves by age group.

**FIGURE 2:**
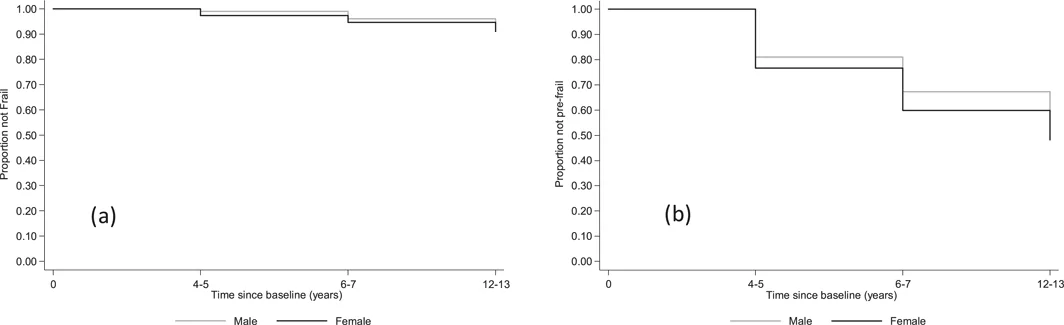
Frailty (2a) and pre‐frailty (2b) survival curves by sex.

**FIGURE 3:**
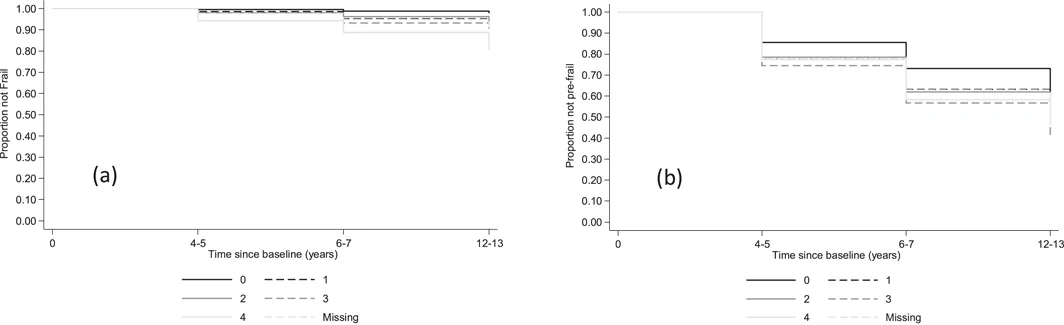
Frailty (3a) and pre‐frailty (3b) survival curves by allostatic load.

Figures 1, 2 and 3 show survival curves for frailty and prefrailty by age group, sex and allostatic load, respectively. The results of log rank tests for difference indicate that the differences in survival curves for frailty are significantly different by age category (Figure 1a, *p* < 0.001), sex (Figure 2a, *p* = 0.008) and allostatic load (Figure 3a, *p* < 0.001). Similarly, the results of log rank tests for difference indicate that the differences in survival curves for prefrailty are significantly different by age category (Figure 1b: *p* < 0.001), sex (Figure 2b: *p* = 0.002) and allostatic load (Figure 3b, *p* < 0.001). For both outcomes, older participants, females and those with high allostatic load are at higher risk.

### 3.3 Discrete Time Event History Analysis

Tables 4 and 5 show the discrete time event history analysis for frailty and prefrailty, respectively. Having an increasing allostatic load score is indicative of a greater risk of both frailty and pre‐frailty for each model, but in the final model having an allostatic load score of four remained the sole allostatic load predictor for frailty, and allostatic load score of three for pre‐frailty.

**TABLE 4:** Discrete time logit model for frailty.

	Model 1: Allostatic load & time			Model 2: Allostatic load, demographic characteristics & time			Model 3: Allostatic load, socioeconomic characteristics & time			Model 4: Allostatic load, health characteristics & time			Model 5: Allostatic load, social support characteristics & time			Model 6: Full model		
	OR	SE	*p*‐value	OR	SE	*p*‐value	OR	SE	*p*‐value	OR	SE	*p*‐value	OR	SE	*p*‐value	OR	SE	*p*‐value
Allostatic load																		
(0)																		
1	4.2	1.71	0.001**	2.7	1.11	0.019*	3.9	1.63	0.001**	3.7	1.52	0.002**	4.0	1.66	0.001**	2.2	0.93	0.060
2	3.6	1.49	0.002**	2.3	0.98	0.047*	3.2	1.32	0.006**	2.9	1.24	0.011*	3.5	1.45	0.003**	1.6	0.71	0.247
3	5.8	2.44	< 0.001***	3.9	1.70	0.001**	4.8	2.05	< 0.001***	4.3	1.88	0.001**	5.6	2.36	< 0.001***	2.1	0.93	0.108
4	11.5	5.03	< 0.001***	8.1	3.60	< 0.001***	9.9	4.36	< 0.001***	7.6	3.59	< 0.001***	11.0	4.82	< 0.001***	3.3	1.62	0.013*
Missing	4.8	1.94	< 0.001***	3.4	1.40	0.003**	4.3	1.74	< 0.001***	3.9	1.61	0.001**	4.7	1.91	< 0.001***	2.2	0.91	0.067
Sex																		
(Male)																		
Female				1.5	0.23	0.017*										1.4	0.23	0.067
Age (years)				1.2	0.01	< 0.001***										1.2	0.01	< 0.001***
Retirement Status																		
Not retired																		
Retired				0.8	0.15	0.270										0.8	0.15	0.335
Education																		
(No qualifications)																		
Some qualifications							0.5	0.07	< 0.001***							0.8	0.13	0.110
Wealth																		
(Low)																		
Medium to high							0.5	0.09	< 0.001***							0.6	0.14	0.039*
Smoking Status																		
(Not a current smoker)																		
Current smoker										1.1	0.26	0.637				1.6	0.39	0.065
BMI										1.0	0.02	0.220				1.1	0.02	0.001**
Alcohol Consumption																		
(Less than 3 times a week)																		
At least 3 times a week										0.6	0.10	0.002**				0.7	0.12	0.030*
Cognitive Function										0.9	0.01	< 0.001***				0.95	0.02	0.003**
Social Integration																		
(High)																		
Medium													1.4	0.26	0.075	1.4	0.27	0.102
Low													1.1	0.26	0.691	0.9	0.23	0.747
Missing data													1.2	0.31	0.588	0.9	0.27	0.786
Social Support																		
(High)																		
Medium													1.1	0.22	0.496	1.5	0.32	0.032*
Low													1.1	0.26	0.612	1.7	0.41	0.038*
Missing													1.7	0.43	0.048*	1.5	0.41	0.103
Time since baseline																		
(4–5 years (wave 4: 2008–09))																		
6–7 years (wave 6: 2012–13)	1.5	0.28	0.015*	1.7	0.32	0.004**	1.6	0.29	0.008**	1.7	0.30	0.004**	1.5	0.28	0.016*	1.8	0.34	0.001**
12–13 years (wave 8: 2016–17)	1.8	0.33	0.002**	2.4	0.46	< 0.001***	1.9	0.36	0.001**	2.1	0.40	< 0.001***	1.8	0.33	0.002**	2.7	0.54	< 0.001***
Constant	< 0.1	< 0.01	< 0.001***	< 0.1	< 0.01	< 0.001***	< 0.1	< 0.01	< 0.001***	0.1	0.07	0.001**	< 0.1	< 0.01	< 0.001***	< 0.1	< 0.01	< 0.001***

*Note:* Reference categories shown in parenthesis.

Abbreviations: OR, Odds Ratio; SE, Standard Error.

^*^ *p* < 0.05.

^**^ *p* < 0.01.

^***^ *p* < 0.001.

**TABLE 5:** Discrete time logit model for prefrailty.

	Model 1: Allostatic load & time			Model 2: Allostatic load, demographic characteristics & time			Model 3: Allostatic load, socioeconomic characteristics & time			Model 4: Allostatic load, health characteristics & time			Model 5: Allostatic load, social support characteristics & time			Model 6: Full model		
	OR	SE	*p*‐value	OR	SE	*p*‐value	OR	SE	*p*‐value	OR	SE	*p*‐value	OR	SE	*p*‐value	OR	SE	*p*‐value
Allostatic load																		
(0)																		
1	1.4	0.16	0.003**	1.3	0.15	0.037*	1.4	0.16	0.004**	1.3	0.15	0.015*	1.4	0.16	0.004**	1.2	0.14	0.113
2	1.5	0.17	< 0.001***	1.4	0.16	0.009**	1.5	0.17	0.001**	1.4	0.16	0.010*	1.5	0.17	< 0.001***	1.2	0.14	0.149
3	1.9	0.24	< 0.001***	1.8	0.23	< 0.001***	1.9	0.24	< 0.001***	1.6	0.22	0.001**	1.9	0.25	< 0.001***	1.4	0.19	0.022*
4	1.7	0.29	0.002**	1.4	0.25	0.038*	1.6	0.28	0.005**	1.2	0.23	0.242	1.7	0.29	0.003**	0.97	0.19	0.880
Missing	1.5	0.17	< 0.001***	1.4	0.16	0.002**	1.5	0.17	< 0.001***	1.4	0.16	0.004**	1.5	0.17	< 0.001***	1.2	0.14	0.108
Sex																		
(Male)																		
Female				1.3	0.09	< 0.001***										1.3	0.09	< 0.001***
Age (years)				1.04	0.01	< 0.001***										1.04	0.01	< 0.001***
Retirement status																		
Not retired																		
Retired				1.1	0.09	0.248										1.1	0.09	0.170
Education																		
(No qualifications)																		
Some qualifications							0.8	0.06	0.013*							1.04	0.09	0.647
Wealth																		
(Low)																		
Medium to high							0.8	0.10	0.107							0.9	0.12	0.653
Smoking Status																		
(Not a current smoker)																		
Current smoker										0.97	0.10	0.760				1.1	0.12	0.300
BMI										1.02	0.01	0.006**				1.04	0.01	< 0.001***
Alcohol Consumption																		
(Less than 3 times a week)																		
At least 3 times a week										0.8	0.05	0.002**				0.8	0.06	0.014*
Cognitive Function										0.97	0.01	< 0.001***				0.99	0.01	0.154
Social Integration																		
(High)																		
Medium													1.03	0.08	0.693	1.03	0.09	0.687
Low													1.04	0.10	0.688	1.1	0.11	0.532
Missing data													0.9	0.11	0.399	0.95	0.12	0.701
Social Support																		
(High)																		
Medium													1.1	0.09	0.427	1.2	0.10	0.072
Low													1.2	0.12	0.044*	1.3	0.13	0.004**
Missing													1.1	0.13	0.693	0.95	0.12	0.693
Time since baseline																		
(4–5 years (wave 4: 2008–09))																		
6–7 years (wave 6: 2012–13)	0.9	0.07	0.204	0.96	0.07	0.559	0.9	0.07	0.271	0.9	0.07	0.397	0.9	0.07	0.194	0.97	0.07	0.717
12–13 years (wave 8: 2016–17)	0.9	0.08	0.302	1.03	0.10	0.766	0.9	0.08	0.428	0.96	0.09	0.659	0.9	0.08	0.303	1.1	0.10	0.498
Constant	0.2	0.02	< 0.001***	< 0.1	< 0.01	< 0.001***	0.3	0.04	< 0.001***	0.3	0.08	< 0.001***	0.2	0.02	< 0.001***	< 0.1	< 0.01	< 0.001***

*Note:* Reference categories shown in parenthesis.

Abbreviations: OR, Odds Ratio; SE, Standard Error.

^*^ *p* < 0.05.

^**^ *p* < 0.01.

^***^ *p* < 0.001.

For frailty, in the fully adjusted model (Table 4, model 6), being of greater age (*p* < 0.001, OR = 1.2), having a higher BMI (*p <* 0.001, OR = 1.1), having medium (*p* = 0.032, OR = 1.5) or low (*p* = 0.038, OR = 1.7) social support, and greater time since baseline (6–7 years: *p* = 0.001, OR = 1.8; 12–13 years: *p* < 0.001, OR = 2.7) all significantly increased the likelihood of developing frailty. Being in the medium‐high wealth bracket (*p* = 0.039, OR = 0.6), having higher cognitive function (*p* = 0.003, OR = 0.95) and having more frequent alcohol consumption (over three times a week, *p* = 0.030, OR = 0.7) significantly decreased the likelihood of developing frailty.

For pre‐frailty, in the fully adjusted model (Table 5, model 6), being female (*p* < 0.001, OR = 1.3), of greater age (*p* < 0.001, OR = 1.04), having a higher BMI (*p* < 0.001, OR = 1.04), and having low social support (*p* = 0.004, OR = 1.3) significantly increased the likelihood of developing pre‐frailty, whilst having more frequent alcohol consumption (over three times a week, *p* = 0.014, OR = 0.8) significantly decreased the likelihood of developing pre‐frailty.

Interaction terms were tested between sex and allostatic load and age and allostatic load but found not to be statistically significant. This indicates no evidence that the association between allostatic load and frailty varied by sex or age.

A higher allostatic load score at baseline was indicative of a greater risk of both frailty (Table 4, model 1) and pre‐frailty (Table 5, model 1). When allostatic load, demographic characteristics, social characteristics, health characteristics and social support characteristics were all included in the model, an allostatic load score of four at baseline remained a predictor for frailty (Table 4, model 6), and an allostatic load score of three at baseline remained a predictor for pre‐frailty (Table 5, model 6).

## 4 Discussion

The present study set out to understand whether allostatic load could serve as a predictor of frailty onset up to 12 years ahead of a frailty designation. Allostatic load at baseline, in adults as young as 50–59 years old, was predictive of both frailty onset and pre‐frailty onset when controlling for a host of demographic, behavioural, and social variables associated with health. The association between allostatic load and frailty onset was stronger than between allostatic load and pre‐frailty onset, whilst higher age, higher BMI, less frequent alcohol consumption were all significant predictors for both pre‐frailty and frailty onset. Lower social support was an additional predictor of frailty onset.

The main finding of this study is that allostatic load score is predictive of frailty development, even after accounting for demographic, socio‐economic, health and social support. Over the observation period, 6% of the baseline sample were observed to develop frailty and 37% were observed to develop pre‐frailty, although these proportions should be interpreted in the context of loss to follow‐up and incomplete outcome data. Previous research (Ding et al. 2017; Shi et al. 2023) had suggested that allostatic load and frailty are related, but did not exclude existing baseline frailty status, suggesting it might be due to co‐existence rather than causal. The present study shows that allostatic load predicts the transition to frailty in non‐frail populations and that this now extends over a 12‐year period populations. In addition, compared with (Ding et al. 2017; Gruenewald et al. 2009; Karlamangla et al. 2002), this study extends the age to those aged 50 or higher, although our included sample was relatively young older adults (aged 63 ± 8 years). In healthy individuals at baseline without any frailty indicators, a high allostatic load score predicts increased risk of developing frailty within 12 years. Thus, allostatic load is not only related to frailty, it precedes and may be a contributing factor to frailty onset.

Whilst an allostatic load score of 4 increased the risk of frailty onset, pre‐frailty onset was predicted by an allostatic load score of 3, and not 4. Firstly, this suggests that a moderate level of allostatic load (scores of 1–2) were not associated with significantly greater odds of developing frailty. That only the highest score of 4 increased the risk of developing frailty suggests that dysregulation in the biological systems of allostatic load (immune, metabolic, and cardiovascular systems) plays a role in the development of physical frailty over a considerable timespan (Duignan and Dutton 2025). Indeed, the ageing process is typically considered to be moderate in the included population (63 ± 8 years old at baseline) (Walsh et al. 2023). This would offer potential for allostatic load score to function as a very early form of intervention indication. Secondly, that pre‐frailty onset is not linked to allostatic load score of 4 is unexpected. Tentatively, it could indicate that progression of allostatic load score of 4 is rapid and comprises less than 4 years, and therefore may bypass the pre‐frailty stage. Although it should also be acknowledged that our baseline sample is likely biased: those with an allostatic load score of 4 would likely have had pre‐frailty at baseline and thus not eligible for inclusion. Thus, making it more likely that they were categorised as frail in the next included wave, or lost to follow up.

Demographic, socio‐economic, health and social support factors overlapped with both allostatic load and frailty onset, and have previously been shown to link to both allostatic load and frailty (Duignan and Dutton 2025). For both frailty and pre‐frailty onset, being of greater age, having a higher BMI, having less frequent alcohol consumption, having low social support were all associated with a higher likelihood of developing (pre‐) frailty in the final accumulated model. Age (Fried et al. 2001) and low social support (Dou et al. 2025) have been reported previously, but for BMI (Jayanama et al. 2022) and alcohol consumption (Guo et al. 2023; Kojima et al. 2017), this seems more complex and less clear.

Being in a higher wealth bracket and having higher cognitive function, were significant protective factors for frailty onset, but not pre‐frailty onset. Similarly, a greater time since baseline predicted frailty onset but not pre‐frailty onset. A likely explanation is that pre‐frailty is a state of one or two deficits, which might not reflect the ageing process as clearly as frailty does (Sezgin et al. 2022). Furthermore, our analyses indicated that 3% of the population became frail without an observed pre‐frailty classification (over a 4‐year time period). That 97% were pre‐frail prior to frail indicates that the frailty process is typically a slower decline than 4 years and can be reversed (Apóstolo et al. 2018). This would then offer pre‐frailty screening as a second time point for intervention. Finally, that being female was a risk factor for developing pre‐frailty, but not frailty, might suggest that women have a longer pre‐frailty stage (Mielke et al. 2022). This also is akin to the previously observed paradox that women have a higher prevalence of frailty, but also live longer (Gordon et al. 2017), suggesting that females tolerate (pre‐)frailty better.

The reduced impact on allostatic load score on (pre‐)frailty onset when accounting for both modifiable and non‐modifiable factors (for demographic, socio‐economic, health and social support) suggests that allostatic load and frailty may act via similar mechanisms related to ageing well. It also highlights the complex inter‐connected nature of these overlapping constructs. The fully adjusted model (model 6) showed fewer and lower odds ratios for allostatic load compared with the less complex models (model 2–5), and possibly highlights the risk of overadjustment. Nevertheless, some of the factors associated with allostatic load and frailty may be modifiable to some extent (Duan et al. 2024) and should thus be considered as potential targets for improvement. Recently, it was shown that depression can mediate the effect of allostatic load on frailty progression, although this was mainly for adults younger than 60 years (Azizzadeh et al. 2026). It is of interest to note that social support was a factor with relatively large odds ratios. Indeed, a recent systematic review (Dou et al. 2025) reported that 4 out of 5 included longitudinal studies showed that increased social support reduced frailty risk. Our study hints that this might indeed be a factor in developing frailty, in line with a recent study suggesting that social participation can reduce the negative impact of allostatic load on healthy ageing (Sahab et al. 2025). Since social support is modifiable this thus warrants focus as early intervention.

The number of frailty occurrences (*N* = 186 in total) was below the threshold (120 occurrences in each age bracket) to warrant exploring age‐related differences separately for each age category. For pre‐frailty, where occurrences were sufficient (*N* = 1215), there was no significant interaction effect across age, or sex in the Discrete Time Logit Model. This indicates that the association between allostatic load and frailty does not vary by age and sex. It implies that allostatic load predicts (frailty regardless of age and sex, which is beneficial as it can be applied without needing to take either age or sex into account). In addition, statistically significant associations between age and sex with both frailty and pre‐frailty in the survival curves confirm previous research that older age and being female increases the risk of (pre‐)frailty (Gordon et al. 2017). Combined, it suggests that using allostatic load as the biological process underpinning the prediction of frailty is a more suitable approach than a prediction based on age and/or sex (Lang et al. 2009).

One limitation of using the ELSA data for secondary data analyses was that missing data was highly prevalent. Sample loss was 60% for frailty and 46% for pre‐frailty and impacted our sample's representativeness of the population. Consequently, our final sample was largely aged under 70 years, and thus our results should be interpreted in that age bracket. It was also relatively highly educated and medium‐to‐high income, whilst not taking into account ethnicity (Olowokere et al. 2025). Secondly, inclusion criteria of not having any factor of frailty also meant our study is primarily applicable to a healthy population at baseline, which means it is of increased value in prevention strategies but less applicable to (pre‐)frail individuals. Nevertheless, our study showed that even in this healthy population, allostatic load predicts the risk of developing frailty. The use of population sample‐based cut offs (e.g., quartiles, quintiles) rather than pre‐defined cut offs might also have impacted our comparison with other studies, although these studies often also used population specific cut off values (Ding et al. 2017; Sanders et al. 2017). Finally, despite pre‐registration and relying on previously published methodology, several amendments had to be made to the methodology because the data were not suitable for the original analysis plan. The most notable amendment was using seven, rather than eight, allostatic load indicators due to the removal of Hba1c, but the conceptualisation of allostatic load was still able to assess multiple systems and utilised the remaining markers available across waves reported in other robust studies using these data (Richards et al. 2023). The conceptualisation of allostatic load is a continuing debate in the field, with some consensus documents being produced that provide specific guidance on allostatic load quantification and modelling (see: Carbone et al. 2022; McCrory et al. 2023), however these guidelines were not practicable with the constraints of the ELSA dataset. However, it is not likely to have impacted the results, and any deviations have been transparently reported.

Practical recommendations comprise that screening for high allostatic load in currently non‐frail older adults may be an appropriate method to predict future frailty trajectories over a 12‐year period. A recent study indicated that it is not only physical health that is impacted by allostatic load, but also risk of depression (Zhao et al. 2025). Thus, allostatic load can, even in a currently non‐frail population, predict the risk of frailty onset, paving the way for early intervention and prevention strategies. The strength of the relationship was however smaller when co‐variates were included, indicating that there is overlap in the mechanisms. Social support may represent one potentially modifiable factor that can help prevent high allostatic load leading to frailty. Future research could explore whether changes in social support can impact positively on both allostatic load and frailty.

In conclusion, allostatic load was prospectively associated with the transition to (pre‐)frailty and should be considered as a possible screening tool for future frailty risk. Both modifiable and non‐modifiable factors should be considered when developing strategies to help prevent or delay the onset of frailty, with social support representing one potentially modifiable factor for further investigation.

## Ethics Statement

Ethical approval for ELSA was granted by the Multicenter Research and Ethics Committee.

## Conflicts of Interest

The authors declare no conflicts of interest.

## Data Availability

The data that support the findings of this study are openly available in English Longitudinal Study of Ageing (ELSA) at http://doi.org/10.5255/UKDA‐Series‐200011.
